# Efficacy of Intralesional Measles Mumps Rubella Vaccine in the Treatment of Verruca Vulgaris: An Interventional Study

**DOI:** 10.7759/cureus.34338

**Published:** 2023-01-29

**Authors:** Ashma S Surani, Raghavon UN, Yogesh Marfatia

**Affiliations:** 1 Dermatology, Byramjee Jeejabhoy (BJ) Medical College and Civil Hospital, Ahmedabad, IND; 2 Dermatology, Pandit Deendayal Upadhyay (PDU) Medical College and Hospital, Rajkot, IND; 3 Dermatology, Smt. BK Shah Institute and Research Centre, Vadodara, IND

**Keywords:** immunotheraphy, intra-lesional, human papilloma virus, viral warts, measles mumps rubella vaccine

## Abstract

Background

A wart is a mucocutaneous illness caused by the growth of HPV-infected skin or mucosal cells. Intralesional immunotherapy makes use of the immune system's ability to identify injected antigens, which might cause a delayed-type hypersensitivity reaction not just to the antigen but also to the wart virus. This, in turn, improves the immune system's ability to identify and eliminate HPV not just at the treated wart but also at distant places, as well as prevent recurrences.

Aims and objectives

To study the efficacy of the intralesional measles, mumps, and rubella (MMR) vaccine in verruca vulgaris and its side effects.

Materials and methods

Interventional research with a 94-case sample size was conducted over a period of seven months. A volume of 0.3 ml of the MMR vaccination was reconstituted with sterile water and injected into the largest wart at three-week intervals until complete clearance or for a maximum of three treatments. Following a six-month observation period, patients were evaluated to look for recurrence, and the degree of response was categorized as total, partial, or none at all.

Results

The youngest case included in the study was of age 10 years and the eldest case was of 45 years. The mean age was 28.22± 10.98. Of 94 patients, 83 (88.3%) were men and 11 (11.7%) were women. Complete remission was reported in 38 (40.42%) cases, a partial response in 46 (48.94%) cases, and no response in 10 (10.63%) cases. All 38 patients who showed complete clearance had a duration of warts in six months or less. The pain was a universal complaint (100%) after each visit followed by bleeding at 25.53%. Flu-like symptoms were noted in three cases after the first dose and two cases after the second dose, while urticaria was seen in one case during all visits. Cervical lymphadenopathy was observed in two cases after the first dose. Erythema multiforme minor was seen only in one patient after the first dose.

Conclusion

Intra-lesional MMR vaccine therapy proved to be a simple and safe treatment option in cases having multiple warts. The response rate may increase if a higher concentration of vaccine (0.5ml) and additional doses (maximum of five doses) are given.

## Introduction

Mucocutaneous warts are a disease that develops as a result of the proliferation of infected skin or mucosal cells with the human papilloma virus (HPV) [1].

Mucocutaneous warts are frequently asymptomatic and seek patients' attention due to the aesthetic issues they pose. This condition should be addressed since it has a significant impact on patients' quality of life. The immune system, the cell-mediated immunity (CMI) has a role in HPV proliferation through the stimulation of cytokines. Intra-lesional immunotherapy makes use of the immune system's ability to identify injected antigens, which may provoke a delayed-type hypersensitivity reaction not just to the antigen but also to the wart virus. This, in turn, improves the immune system's ability to identify and eliminate HPV not just at the treated wart but also at distant locations, as well as prevent recurrences [2].

Current therapeutic modalities for viral warts are mostly ablative and are limited by high recurrence rates besides being unsuitable for numerous lesions. Intralesional antigens used in immunotherapy include the measles, mumps, and rubella (MMR) vaccine, tuberculin purified protein derivative (PPD), Bacillus Calmette-Guerin (BCG) vaccine, Mycobacterium w (Mw) vaccine, Candida albicans antigen, and others [3-8]. Because of the high occurrence of warts in many populations, particularly children, through this study an attempt is made to evaluate the role of immunotherapy by MMR vaccine injection in cutaneous warts.

## Materials and methods

Over a period of seven months, an interventional study involving 94 cases was carried out in a tertiary care center to know the therapeutic outcomes and efficacy of the MMR vaccine in viral warts. At three-week intervals, a volume of 0.3 ml of MMR vaccine was reconstituted with sterile water and injected into the largest wart until complete remission, or for a maximum of three treatments. Patients were examined after a six-month observation period to look for recurrence, and the degree of response was classified as total, partial, or none at all. Prior approval from the Ethics committee was taken. A total of 100 patients with veruca vulgaris (common warts) attending the outpatient department (OPD) of dermatology and giving written consent were enrolled, out of which six cases were lost to follow-up. Hence 94 cases were studied.

Inclusion criteria

Healthy male and female (without childbearing potential) aged 10 to 45 years with a clinical diagnosis of verruca vulgaris, no concurrent systemic, or topical treatment of warts within the past two weeks attending the dermatology OPD, and cases with multiple common warts. Multiple warts were defined as more than five common warts at a single anatomic site or one or more common warts at more than one anatomical site. 

Exclusion criteria and methods

Cases with fever or signs of any inflammation or infection, systemic and/or cutaneous diseases, asthma or allergic skin conditions, meningitis, or convulsions in the past, history of any live vaccine or immunoprophylactic drug within the last three months, and immunocompromised cases were excluded from the study. After informed consent, detailed history was taken followed by a proper cutaneous examination. 

MMR vaccine was reconstituted with sterile water and a volume of 0.3 ml was injected with an insulin syringe into the base of the largest wart. This was repeated every three weeks until complete clearance of all warts or for a maximum of three treatments. The response was graded as complete, partial, or no response and was followed up after a period of six months to detect recurrence if any.

Friedman test was used to analyze the relationships between variables. A mean rank table was established to assess the final outcome of the study.

## Results

The youngest case included in the study was of age 10 years and the eldest case was of 45 years. The mean age was 28.22± 10.98. Out of 94 cases, 83 (88.3%) were males and 11 (11.7%) were females (Table 1).

**Table 1:** Age & Sex wise distribution of common wart cases

Gender	Age groups of cases (years)			Total cases (n=94) (%)
	≥ 10 and ≤ 20	21-30	31-45	
Male	24	24	35	83 (88.3)
Female	4	0	07	11 (11.7)
Total	28	24	42	94 (100)

Duration of warts was less than or equal to six months in the majority of cases; i.e. 70 out of 94 (74.5%) cases (Table 2).

**Table 2:** Distribution of cases based on the duration of warts

Sr. No.	Duration in months	Number of cases (n=94) (%)
1	1-6	70 (74.5)
2	7-12	19 (20.21)
3	13-18	4 (4.25)
4	19-24	1 (1.06)

Common sites of verruca vulgaris were the face, neck, and hand in 22, 20, and 21 cases respectively (Table 3).

**Table 3:** Distribution of cases based on site of warts

Sr. No.	Sites of warts	Number of patients
1	Scalp	1
2	Face	22
3	Neck	20
4	Hand	21
5	Elbow	2
6	Forearm	2
7	Leg	1
8	Knee	1
9	Face + Neck	13
10	Face + Foot	1
11	Hand + Neck	1
12	Hand + Forearm	2
13	Hand + Face	3
14	Hand + Foot	2
15	Forearm + Foot	2
Total		94

**Figure 1:**
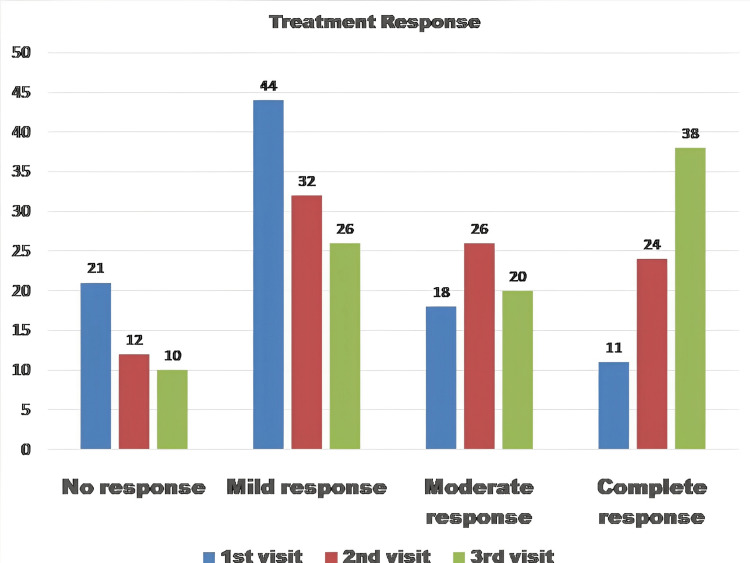
Therapeutic response at the end of the study

The therapeutic response was measured at every follow-up following the MMR vaccine and analyzed at the end of the study (Table 4 and Figure 1) as no response as Grade 1 (no improvement), mild response as Grade 2 (< 50% improvement), moderate response as Grade 3 (> 50% improvement), and complete response as Grade 4.

**Table 4:** Therapeutic response at the end of study period n- Total number of patients

Treatment response	First visit		Second visit		Third visit	
	n	%	n	%	n	%
Grade 1 (no response)	21	22.3	12	12.8	10	10.6
Grade 2 (< 50% / mild response)	44	46.8	32	34.0	26	27.7
Grade 3 (>50% / moderate response)	18	19.1	26	27.7	20	21.3
Grade 4 (complete response)	11	11.7	24	25.5	38	40.4
Total	94	100.0	94	100.0	94	100.0

The Friedman test revealed that the results were significant with the MMR vaccine in the case of multiple wart patients. The mean rank significantly increased with each visit (after vaccination), χ2 = 82.17, p-value = 0.001. The median indicates the treatment response was higher on the third visit (median =3) and the least was on the first visit (median = 2). When the number of MMR vaccine injections was higher, the response was greater (Table 5). 

**Table 5:** Descriptive study table

	Percentiles			Mean rank	Chi-square (P-value)
	25th	50th (Median)	75th		
Visit one	2.00	2.00	3.00	1.53	82.17 (0.001)
Visit two	2.00	3.00	4.00	2.09	
Visit three	2.00	3.00	4.00	2.38	

Sensations of pain were a universal complaint (100%) during all visits followed by bleeding in 25.53%. Flu-like symptoms were seen in three cases after the first dose and in two cases after the second dose. while urticaria was seen in one case during all visits. Cervical lymphadenopathy was observed in two cases after the first dose. Erythema multiforme minor was seen only in one patient after the first dose (Table 5).

**Table 6:** Side effects of MMR vaccine

Sr. No.	Side effects	Number of cases
1	Pain	94
2	Bleeding	24
3	Flu-like symptoms	03
4	Urticaria	01
5	Cervical lymphadenopathy	02
6	Erythema multiforme minor	01

An 18-year-old male patient with verruca vulgaris on the dorsum of the hand (bilaterally) presented to the dermatology OPD after seven days of the first dose of MMR vaccine with multiple target lesions on B/L palms. There were no other associated complaints. No abnormality of the mucus membrane was noted. HSV serology was negative. The patient was diagnosed with Erythema multiforme minor and managed symptomatically. Lesions resolved within seven days. No similar complaint was present after the subsequent two doses.

Out of the 11 patients having a partial response, two patients (4.37%) had a total recurrence of lesions. While nine patients (19.56%) had a partial recurrence. No recurrence was observed in the patients with complete clearance.

**Figure 2:**
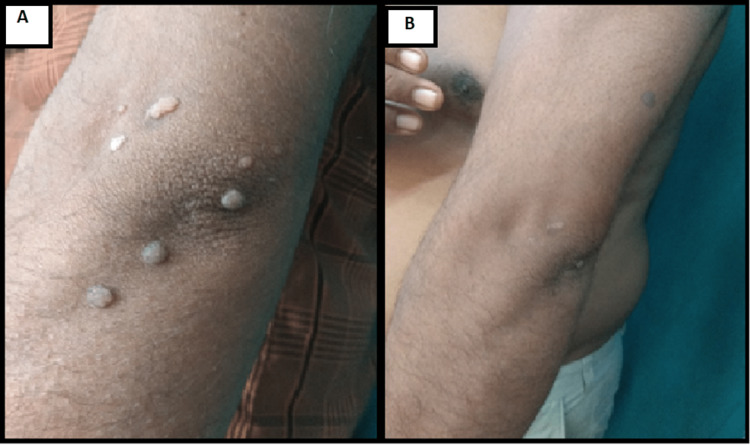
A 32-year-old male with multiple warts for six months showed complete response after a single dose of MMR vaccine (A) before and (B) after therapy

**Figure 3:**
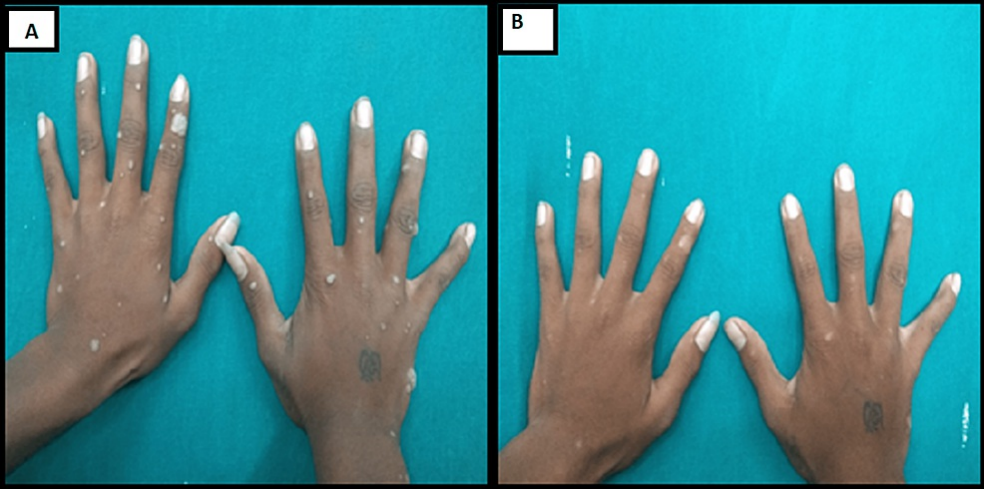
A 16-year-old-male with multiple warts for one month showed complete response after three doses of MMR vaccine (A) before and (B) after therapy

**Figure 4:**
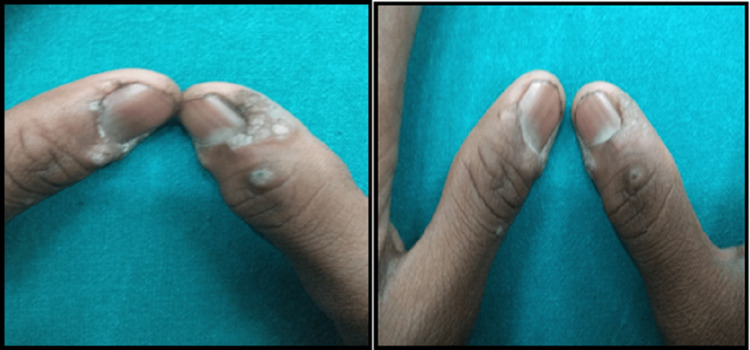
An 18-year-old male with warts for four months showed partial response after three doses of MMR vaccine (A) before and (B) after therapy

Results of a few patients are shown in the following figures with before and after therapy clinical pictures (Figures 2-4)

## Discussion

A plethora of treatments for common warts demonstrates that no one treatment has shown to be 100% effective, and the majority of them remain unsatisfactory. The unrestrained proliferation of warts in HIV-infected patients, as well as a significant epidermal and dermal influx of CD4 + lymphocytes in spontaneously regressing warts, suggests that the immune system, particularly cell-mediated immunity, plays a role in the pathogenesis and persistence of warts [9,10]. This proposed intralesional immunotherapy activates cell-mediated and humoral immunity, resulting in the clearance of intralesionally treated and distant warts with varying degrees of efficacy [9-12].

In the present study, most of the patients belonged to the adult age group. Face, hands, and neck involvement were commonly seen in our study group might be because of more exposure and susceptibility to trauma, pricks, and inoculation. In our study, there were 83 (88.3%) males and 11 (11.7%) females receiving MMR immunotherapy, with a male predominance. Males were more affected than females, perhaps due to outdoor working conditions. We document the effectiveness of intralesional injection of MMR vaccine in common warts.

 

Table 7 shows a comparison of the response to the MMR vaccine in similar studies [13-17]. The present study showed a complete response in 38 out of 94 cases while the study by Agrawal et al. had a complete response in 18 (60%) out of 30 patients. The similarity between these two studies was the number of doses i.e. three doses, whereas the difference was the sample size [16]. Similarly, a study conducted by Saini et al. had complete responses from 40 (46.5%) out of 86 patients [15]. The study by Nofal et al. and Chauhan et al. utilized five doses of MMR vaccine and had a complete response in 81.4% and 82.4% respectively [13-14]. Thus the lower response in the present study has a correlation with the number of doses. 

**Table 7:** Comparison of the response to MMR vaccine in similar studies Dosage schedule: * 0.3 ml intralesional MMR three-week intervals for a maximum of three doses. **0.5 ml intralesional MMR at two-week intervals for a maximum of five doses.

RESPONSE TO MMR	*Present study (N=94)	**Nofal et al. [13] (N=70)	**Chauhan et al. [14] (N=51)	*Saini et al [15] (N=86)	*Agrawal et al. [16] (N=30)	**Dhope et al. [17] (N=20)
Complete	38 (40.42%)	57 (81.4%)	42 (82.4%)	40 (46.5%)	18 (60%)	13 (65%)
Partial	46 (48.94%)	7 (10%)	9 (17.6%)	18 (20.9%)	6 (20%)	6 (30%)
No	10 (10.63%)	6 (8.6%)	2 (3.92%)	28 (32.55%)	6 (20%)	1 (5%)

In the present study, pain was a universal complaint but was tolerable and did not extend beyond the time of injection. Similarly, pain was reported in 85.7% and 60% of cases in studies by Nofal et al. [13] and Agrawal et al. [16] respectively.

Cervical lymphadenopathy and erythema multiforme minor were the two distinct side effects only observed in our study compared to other studies.

In our study, there was no recurrence in 38 patients with complete response treated with MMR immunotherapy during the three-month follow-up. This was similar to the studies done by Nofal et al. [13] and Zamanian et al. [11] where no relapse was seen.

## Conclusions

Intralesional MMR vaccine therapy proved to be a simple and safe treatment option in cases having multiple warts. The generation of HPV-directed immunity via the induction of a delayed-type hypersensitivity reaction to an otherwise unrelated immunogen is one hypothesized mechanism of action of MMR vaccination and other lesional immunotherapeutic antigens. The response rate may increase if a higher concentration of vaccine (0.5ml) and a larger number of doses (maximum five doses) are given. Our findings imply that intralesional MMR might be used as a first-line therapy for numerous warts and as a second-line therapy for warts that are resistant to other treatments.
